# Trabecular Evidence for a Human-Like Gait in *Australopithecus africanus*

**DOI:** 10.1371/journal.pone.0077687

**Published:** 2013-11-05

**Authors:** Meir M. Barak, Daniel E. Lieberman, David Raichlen, Herman Pontzer, Anna G. Warrener, Jean-Jacques Hublin

**Affiliations:** 1 Department of Human Evolution, Max Planck Institute for Evolutionary Anthropology, Leipzig, Germany; 2 Department of Human Evolutionary Biology, Harvard University, Cambridge, Massachusetts, United States of America; 3 School of Anthropology, University of Arizona, Tucson, Arizona, United States of America; 4 Department of Anthropology, Hunter College, New York, New York, United States of America; University of Utah, United States of America

## Abstract

Although the earliest known hominins were apparently upright bipeds, there has been mixed evidence whether particular species of hominins including those in the genus *Australopithecus* walked with relatively extended hips, knees and ankles like modern humans, or with more flexed lower limb joints like apes when bipedal. Here we demonstrate in chimpanzees and humans a highly predictable and sensitive relationship between the orientation of the ankle joint during loading and the principal orientation of trabecular bone struts in the distal tibia that function to withstand compressive forces within the joint. Analyses of the orientation of these struts using microCT scans in a sample of fossil tibiae from the site of Sterkfontein, of which two are assigned to *Australopithecus africanus*, indicate that these hominins primarily loaded their ankles in a relatively extended posture like modern humans and unlike chimpanzees. In other respects, however, trabecular properties in Au africanus are distinctive, with values that mostly fall between those of chimpanzees and humans. These results indicate that *Au. africanus*, like *Homo*, walked with an efficient, extended lower limb.

## Introduction

**Figure 1:**
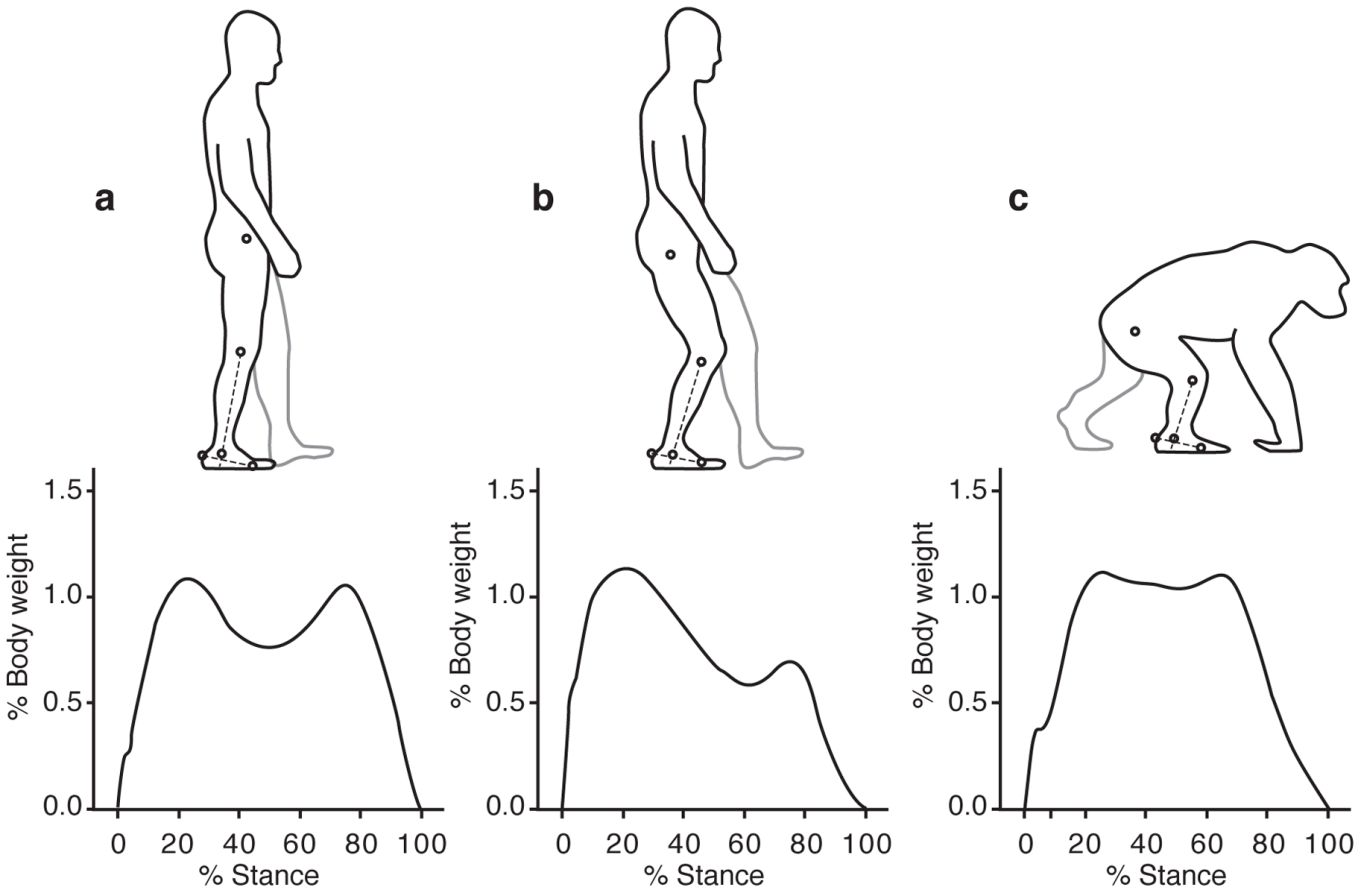
Differences in ankle angle (dashed line) at midstance in humans walking normally (a), with a bent-hip bent-knee gait (b) and chimpanzees walking quadrupedally (c). Note that the ankle is more extended (plantarflexed) during midstance in humans walking normally than chimpanzees walking quadrupedally. The bottom part of the figure shows representative vertical ground reaction force traces plotted as a percentage of body weight over stance duration.

The earliest hominins, *Sahelanthropus*, *Ardipithecus* and *Orrorin*, all have adaptations for upright posture [1]–[3], thus supporting Darwin's conjecture that bipedalism was a key initial derived feature of the hominin lineage [4]. These early hominins, however, may have been facultative bipeds, and the oldest evidence for obligate, non-facultative bipedalism does not appear until 4.2 million years ago in the genus *Australopithecus* [3], [5]–[14]. The nature of australopith bipedalism, however, remains disputed, with most focus on the two best-sampled species: *Au. afarensis* and *Au. africanus*. Some paleoanthropologists infer that these australopiths walked with an efficient, modern gait characterized by relatively extended hips and knees (EHEK) rather than a more bent-hip and bent-knee gait (BHBK) similar to the way chimpanzees walk bipedally (Fig. 1a,1b) [5], [6], [9], [15]–[18]. This view is partly based on simulations and experimental studies of bipedal locomotion, which indicate that EHEK gaits are considerably less energetically costly than BHBK gaits [16], [18]–[20]. Additional support for the hypothesis that australopiths used EHEK gaits comes from an extensive array of anatomical features that are indicative of extended lower limb postures, such as a tibial plateau oriented parallel to the tibiotalar joint surface, the flattened distal contour of femoral condyles, a pronounced lumbar lordosis, and a high femoral carrying angle (i.e., valgus knee) [5], [21], [22].

Two recent studies (which included the fossil distal tibiae we present in the current study, StW 358, 389 and 567) revealed that the distal tibia and ankle joint external morphologies of the genus *Australopithecus* were within the range of the genus *Homo* but different from chimpanzees and gorillas [23], [24]. DeSilva (2009) demonstrated that australopiths resemble humans and differ from chimpanzees and gorillas in having a perpendicularly oriented tibia relative to the horizontal plane of the ankle joint, a square-shaped articular surface of the distal tibia that lacks the wide anterior rim which is found in climbing apes, and a low angle between the axis of rotation and the horizontal plane of the ankle, indicating that these individuals probably possessed a perpendicularly oriented tibia [23]. DeSilva and Throckmorton (2010) also showed that *Australopithecus* possessed a tibial arch angle similar to humans and different from other non-human primates [24]. Other paleoanthropologists, however, consider that retained features which benefit arboreal locomotion in apes such as relatively short hindlimbs, long and curved pedal phalanges, and less coronally-oriented iliac blades (for a complete list see Stern 2000 [25]) compromised australopith walking performance, causing *Au. afarensis* and *Au. africanus* to use a BHBK gait [7], [8], [25]–[28]. Stride lengths from the Laetoli trackway are compatible with either type of gait [29], and while footprint morphology is more consistent with EHEK gaits [9], [12], no single skeletal feature so far documented can reliably and definitively distinguish between EHEK and BHBK gaits.

**Figure 2:**
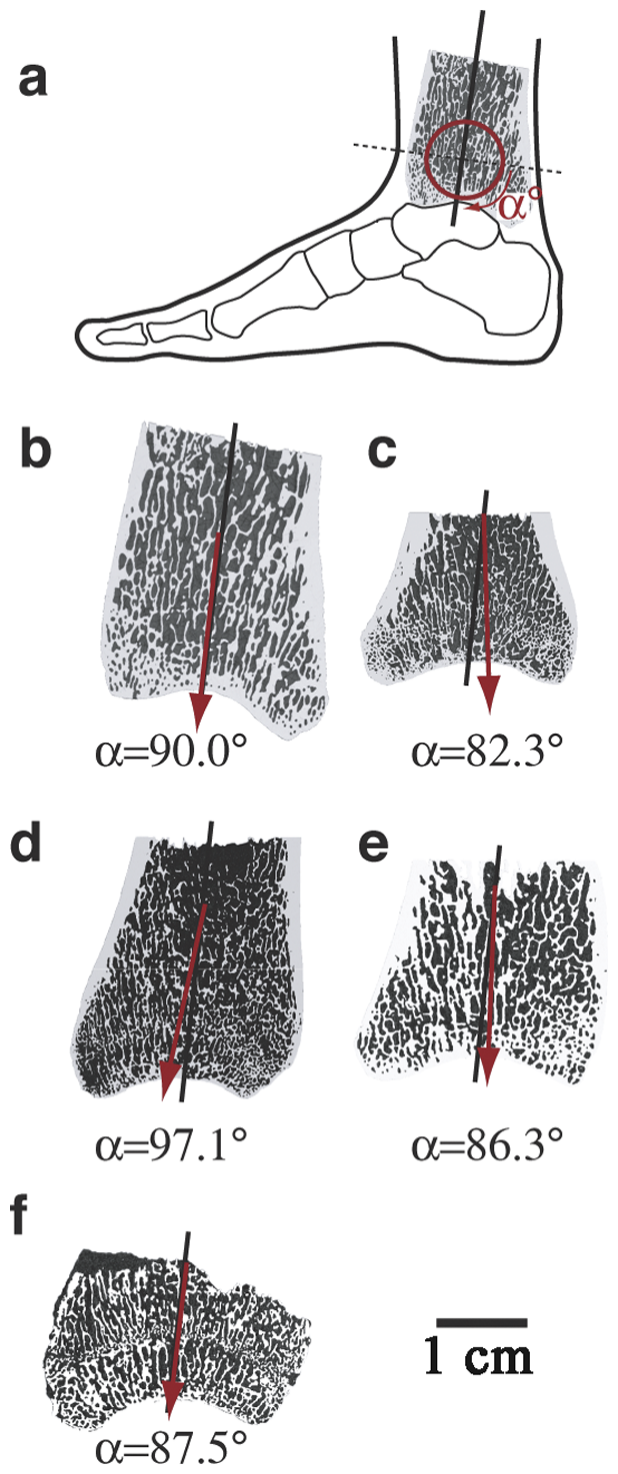
Mid-sagittal views of 2D-PTO in the distal tibia (anterior corresponds to the left side of each bone). Black lines represent the long axis of the bone. The 2D-PTO for each bone was measured as the angle (α) between the 2D-PTO and the normal plane to the long axis of the bone (represented as a horizontal dashed line in Fig. 2a). Red arrows represent the average 2D-PTO for chimpanzees (Fig. 2c, α = 92.3±10.7°) and for humans (Fig 2b, α = 90.0±2.3°)) or the specific 2D-PTO for the fossil samples StW 358 (Fig. 2d), StW 389 (Fig. 2e), and StW 567 (Fig. 2f). Scale bar, 1 cm.

An alternative approach to assess whether early hominins walked with EHEK or BHBK gait is to use the orientation of trabecular struts deep to the articular surface of the hindlimb joints (see Fig. 2a). This strategy takes advantage of Wolff's Law of trabecular orientation, first proposed in 1892, that trabecular struts within joints respond to external loads by preferentially aligning their long axes along the trajectories of peak principal stresses [30]. Despite equivocal results from some comparative studies [31], [32], numerous studies support Wolff's Law both in sub-adults and skeletally mature animals and humans (to name a few [33]–[43]). More importantly, two controlled experiments demonstrated that the relationship between principal trabecular orientation (PTO) and the orientation of peak compressive forces in limb joints during loading is sufficiently accurate and precise to distinguish between individuals that load their joints in slightly different orientations. In one experiment, bipedal birds run for 10 minutes a day (6 days/week) on a 20° inclined treadmill, flexed their knee joints on average 13.7° more than birds run on a flat treadmill (76.3±1.33° and 62.6±3.52° for the birds run on flat and inclined treadmills, respectively, P<0.01), causing a 13.6° shift in the sagittal plane 2D-PTO within the distal femur (P<0.01) [44]. In another experiment, sheep exercised on a flat and 7° inclined treadmill (15 min/day, 6 days/week), altered the angle of the ankle (tibiotalar) joint by 3.6° (124.3±5.3° and 127.9±4.7° for the sheep exercised on flat and inclined treadmills, respectively, P<0.01), leading to a 4.3° shift (P<0.05) in sagittal plane 2D-PTO of the distal tibia medial side [45]. Therefore, even subtle differences in limb orientation during loading can be detected in trabecular bone in the ankle of medium-sized mammals.

Although discussions of BHBK versus EHEK gaits have focused mostly on the hip and knee (two exceptions are [21], [23]), we focus here on the distal tibia of humans and chimpanzees. We do so because trabecular bone in the distal tibia has been shown to be very sensitive to subtle variations in ankle angle during loading in sheep [45], and because the ankle is more extended (plantarflexed) during midstance in humans walking bipedally than chimpanzees walking quadrupedally (Fig. 1), which means that the direction of forces close to the joint surface in the distal tibia should differ between these two species. Since humans have a more extended ankle compared to chimpanzees at midstance during walking (bipedal and quadrupedal for humans and chimpanzees respectively), we postulate that PTO would differ significantly between humans and chimpanzees. We therefore predict that the difference in the sagittal 2D-PTO of the distal tibia between humans and chimpanzees will represent accurately the difference in their ankle joint angle at the midstance phase of their walking cycle. We also predict a significant difference between humans and chimpanzees in their 3D-PTO. Finally, if this hypothesis is not refuted, we would be able to test if *Au. africanus* walked with an EHEK or BHBK gait by comparing the distal tibia PTO from *Au. africanus* and humans.

## Results

### Ankle angles and trabecular orientation in humans and chimpanzees

In order to test the relationship between 2D-PTO and ankle angles we first analyzed data on vertical ground reaction force (GRFv) and tibia orientation in adult chimpanzees (*Pan troglodytes*, n = 3) and a similar-sized sample of adult humans (*Homo sapiens*, n = 6). Both species walked at preferred speeds on a level force plate; chimpanzees walked quadrupedally, and humans were asked to walk with both EHEK and BHBK gaits (Fig. 1). Ankle angle was measured in lateral view as the angle between two lines: from the lateral epicondyle of the femur to the lateral malleolus, and from the posterior tuber calcaneus to the distal head of the 5th metatarsal (Fig. 1, upper part). Because GRFv traces from some chimpanzee and all human trials had a double force peak (Fig. 1, lower part), tibia orientation relative to the long axis of the foot (ankle angle) was averaged over the period of stance when GRFv was greater than 75% of body weight (hereafter termed ‘peak loading’). Mean ankle angle in humans was significantly more extended by 16° when they walked with an EHEK compared to a BHBK gait (85.6°±3.3 and 69.6°±4.3 respectively, P<0.05). When chimpanzees walked quadrupedally, mean ankle angle was 75.2°±3.0, not significantly different to bipedal humans walking with a BHBK gait.

Although chimpanzees sometimes climb and occasionally adopt bipedal postures, quadrupedal walking comprises more than 98% of their locomotor behavior [46]. Therefore trabecular bone in the distal tibiae of chimpanzees is predicted to respond to these external loads by preferentially aligning the long axes of struts along the trajectories of peak principal stresses that are generated during quadrupedal walking around midstance. Thus, if PTO in the distal tibia accurately reflects differences in locomotor posture, we predict an approximately 10° difference in PTO in the distal tibia between humans and chimpanzees.

**Figure 3:**
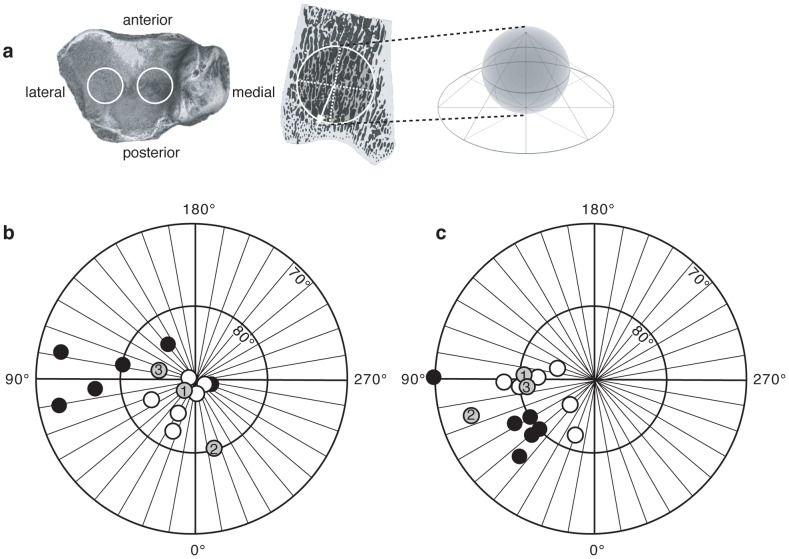
Measurements of 3D-PTO in the distal tibia. (a) Schematic showing location of the lateral and medial VOIs in the distal tibia and how the 3D spheres were visualized in 2D using an equal-angle stereoplot. A stereoplot is a 2D map which is created by projecting points from a surface of a sphere to a tangential plane. (b) The stereoplot projections of the lateral VOI. (c) The stereoplot projections of the medial VOI. Filled circles, chimpanzees; open circles, humans; grey circle 1, StW 358; grey circle 2, StW 389; grey circle 3, StW 567. Angles 0°, 90°, 180° and 270° correspond to the anatomical directions: posterior, lateral, anterior and medial respectively (as given in Fig. 3a). For a detailed account of the 3D-PTO coordinates see Table S1.

We compared microCT scans of distal tibiae from a sample of adult humans (n = 6) and chimpanzees (n = 6). In order to correlate ankle angle to the corresponding PTO in the joint's plane of motion, we determined 2D-PTO in the parasagittal plane from 2D projections using the mean intercept length technique (MIL, see materials and methods for a description of the technique). As predicted by the kinematic data, the 2D-PTO in the distal tibia sagittal plane was inclined significantly more obliquely by 7.7° (P<0.05) in chimpanzees (82.3°±10.7, Fig. 2c) than in humans (90.0°±2.3, Fig. 2b). To further test the correlation between ankle joint loading and PTO, we employed the MIL technique to measure the 3D-PTO in two volumes of interest (VOIs) in the medial and lateral side of the distal tibiae, just deep to the cortex of the joint surface where the talar trochlea contact the distal tibia (the tibial plafond, Fig. 3). As with the 2D analysis, chimpanzees and humans differ significantly in both the medial and lateral VOIs (P<0.05). For a detailed account of the 3D-PTO coordinates see Table S1. Furthermore, Figure 3 reveals a much higher variability in chimpanzees' 3D-PTO, especially in the lateral VOI (see Fig. 3b); these results demonstrate the greater variability in chimpanzee loading of the tibiotalar joint.

### PTO in Sterkfontein tibiae

**Figure 4:**
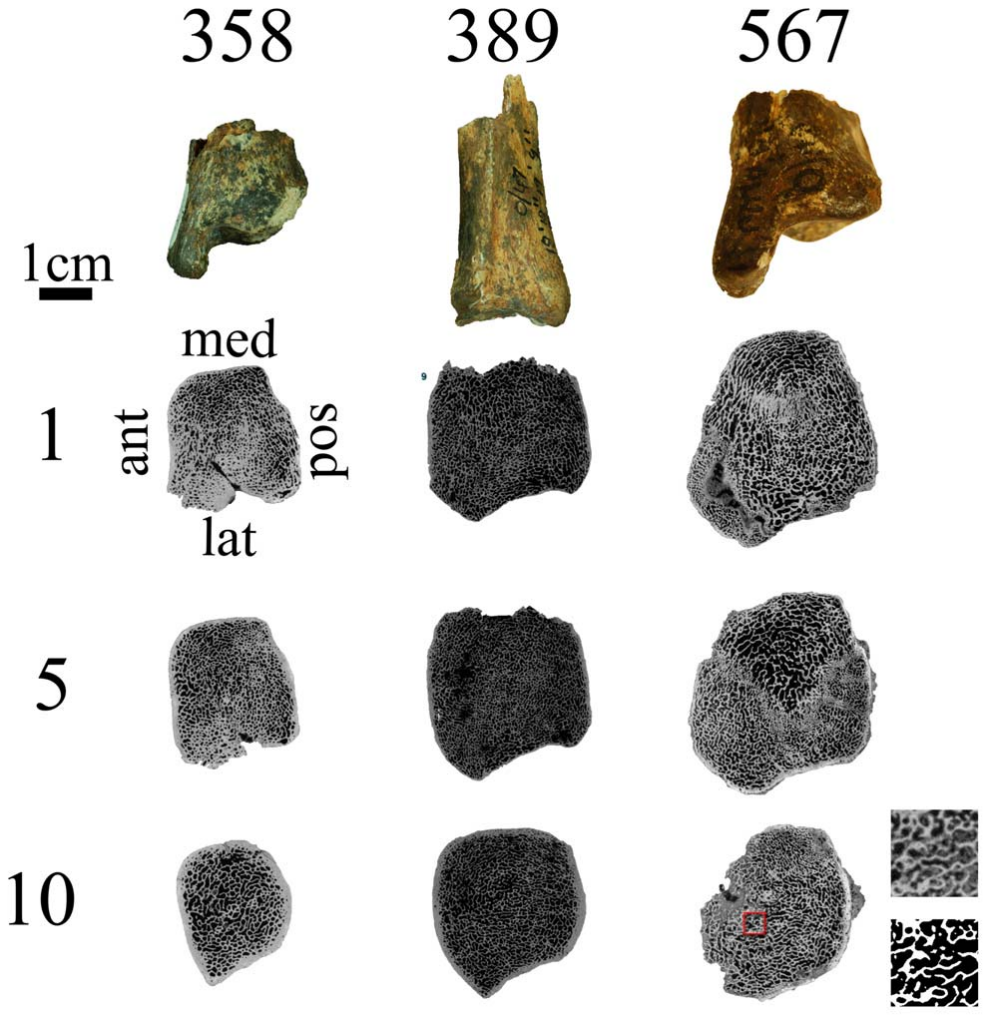
The three Sterkfontein tibiae (StW 358, 389, 567) (upper row) and their trabecular structure 1 mm (2^nd^ row), 5 mm (3^ed^ row) and 10 mm (bottom row) below the cortex, as reveled in transverse slices by the microCT scanning. Scale bar for microCT scans is 1; thus it did not affect our segmentation process (binarization of CT slices). On the bottom right corner of the figure is an inset showing two identical enlargements of an area in StW 567; the upper image is the original, showing typical sedimentation and the bottom image is the same area after segmentation. Note the distinct and clear separation in appearance, consistency and X-ray absorption between the sediments and the actual trabecular structure in the original image.

Given the predictive relationship between ankle angle at peak loading and 2D-PTO in the distal tibia of humans and chimpanzees, we obtained microCT scans of the distal tibia in two *Australopithecus africanus* specimens from Member 4 of Sterkfontein, dated to 2.6–2.8 Ma (StW 358 and StW 389) [23], and one distal tibia from Member 5 of Sterkfontein dated to 1.4–1.7 Ma (StW 567) putatively assigned to early *Homo* [23], [47]. As Figure 4 shows, these bones are well preserved both externally and internally with intact 3D trabecular structure that is detectable using an X-ray source. Using the methods described above for humans and chimpanzees, the 2D-PTO in the sagittal plane is 97.1° in StW 358 (Fig. 2d), 86.3° in StW 389 (Fig. 2e) and 87.5° in StW 567 (Fig. 2f). These values are not significantly different from the orientation in humans (90.0°±2.3, Fig. 2b; P = 0.40; permutation test), but due to the low sample size (n = 3) are also not significantly different from chimpanzees (P = 0.22; permutation test). Because our goal is to test if *Au*. *africanus* walked with an EHEK like modern humans or BHBK, and in order to overcome the fossil small sample size, we tested the combined human and fossil samples (n = 9) versus the chimpanzee samples (n = 6), and the combined chimpanzee and fossil samples (n = 9) versus the human samples (n = 6). While the combined human and fossil samples differed significantly from chimpanzees (P = 0.04; permutation test), the combined chimpanzee and fossil samples were not significantly different from humans (P = 0.22; permutation test). These results indicate that the 2D-PTO of the fossil hominins and humans are similar, but unlike chimpanzees. The same is true for the three-dimensional comparisons in the medial and lateral VOIs (Fig. 3), which reveal no significant difference between fossil hominin and modern human samples (P = 0.19 and P = 0.20 for the medial and lateral VOIs respectively; permutation test), but are significantly different between the combined human and fossil samples chimpanzee samples (P<0.05 for both medial and lateral VOIs).

### Additional trabecular bone properties

MicroCT scans provide the opportunity to compare additional trabecular bone properties between humans, chimpanzees and the fossil hominins, summarized in Table 1. Compared to humans, chimpanzees have significantly more trabeculae per mm (Tb.N), which are less separated (Tb.Sp), thinner (Tb.Th), have a higher connectivity density (ConnD), and a lower degree of anisotropy (DA) in both the lateral and medial VOIs (P<0.01 see Table 1 for details). Although sample sizes are small, the three fossil hominins reveal a distinctive trabecular structure from both humans and chimpanzees (in regards to Tb.N, Tb.Sp, ConnD and DA) and values for their trabecular structural parameters are mostly between those of humans and chimpanzees (Table 1). Remarkably, in all the hominoids taxa (humans, chimpanzees and fossils hominins) the lateral VOI has consistently higher bone volume fraction (BV/TV), Tb.N, Tb.Th and DA (stronger orientation), and lower Tb.Sp compared to the medial VOI. As previous studies have shown that the lateral aspect of the distal tibia in humans is the main load-bearing structure in the tibiotalar joint [48], [49] we would expect to see a corresponding higher trabecular bone volume and a more robust architecture in the lateral VOI. This similarity between chimpanzees and early hominins suggests that the lateral aspect dominancy in tibiotalar joint load-bearing is a primitive trait.

**Table 1:** Trabecular bone properties means, standard deviations (±S.D.) and range (in parentheses).

		*H. sapiens* (n = 6)	*P. troglodytes* (n = 6)	*Au. africanus* (StW 358, 389)	StW 567
Sagittal 2D-PTO		90.0°^*^±2.3 (86.1–92.1)	82.3°^*^±10.7 (72.5–97.3)^#^	97.1°, 86.3°	87.5°
BV/TV	M	23.1±2.7 (18.9–26.4)	26.0±6.2 (17.3–32.4)	34.8, 26.5	30.8
(%)	L	30.2±1.9 (28.1–32.5)	30.5±5.5 (23.5–36.6)	40.5, 27.9	36.7
Tb.N	M	0.90^*^±0.15 (0.73–1.1)	1.41^*^±0.22 (1.1–1.64)	1.36, 1.31	1.23
(1/mm)	L	1.11^*^±0.13 (0.92–1.24)	1.56^*^±0.20 (1.24–1.74)	1.34, 1.37	1.33
Tb.Th	M	0.26^*^±0.02 (0.24–0.29)	0.18^*^±0.03 (0.14–0.21)	0.26, 0.20	0.25
(mm)	L	0.27^*^±0.03 (0.23–0.32)	0.19^*^±0.02 (0.17–0.23)	0.30, 0.20	0.28
Tb.Sp	M	0.82^*^±0.12 (0.63–0.94)	0.55^*^±0.06 (0.49–0.66)	0.58, 0.54	0.61
(mm)	L	0.67^*^±0.11 (0.53–0.81)	0.52^*^±0.06 (0.44–0.62)	0.56, 0.51	0.54
DA	M	2.67^*^±0.55 (2.03–3.3)	1.88^*^±0.14 (1.69–2.08)	2.34, 2.13	1.86
	L	3.16^*^±0.51 (2.61–3.88)	2.27^*^±0.10 (2.13–2.4)	2.42, 2.52	1.98
ConnD	M	4.3^*^±1.4 (2.6–6.4)	12.5^*^±3.1 (8.9–17.3)	4.8, 9.1	7.7
(1/mm^3^)	L	4.5^*^±1.3 (2.8–6.0)	11.5^*^±3.3 (7.7–16.9)	4.0, 8.8	8

BV/TV stands for bone volume fraction, Tb.N for trabeculae per mm, Tb.Th for trabecular thickness in mm, Tb.Sp for trabecular separation in mm, DA for degree of anisotropy and ConnD for connectivity density per mm^3^. L and M stand for lateral and medial VOIs respectively.

Trabecular bone properties differences between humans and chimpanzees were tested for statistical significance using the Wilcoxon rank-sum test. Statistically significant differences between humans and chimps (P<0.01) indicated by *.

# Out of the six chimpanzees only two had 2D-PTO angles larger than 90°, the other four chimpanzees had 2D-PTO angles lower than 83° which is much lower than human 2D-PTO angle range.

## Discussion

The major objective of this study is to test whether trabecular structure in the distal tibia (both 2D-PTO in the sagittal plane and 3D-PTO) reliably predicts known differences in ankle joint angle at the time of peak loading from GRFv during walking in chimpanzees and humans, and to use this signal of loading to infer ankle angles at peak loading in fossil hominin tibiae from Sterkfontein. Our results show that humans' ankle joint angles at peak GRFv are more extended by 10.3° compared to chimpanzees, which corresponds to a 7.7° difference in the sagittal 2D-PTO (Fig. 2b and 2c). Furthermore, 3D measurements of the medial and lateral aspects of the distal tibia demonstrate that the 3D-PTO in humans differs significantly from chimpanzees. These results combined with those from controlled experiments on other species [44], [45] indicate that differences in sagittal 2D- and 3D-PTO in the distal tibia are useful and reliable predictors of joint angle during peak GRFv. In addition, PTO in the distal tibia among the three Sterkfontein fossil hominins is comparable to humans but significantly different from chimpanzees in the 2D sagittal plane as well as in the 3D medial and lateral VOIs (Fig. 3). Although trabecular orientation in these australopith fossils was possibly influenced by loading during climbing, these hominins were unlikely to have climbed more than chimps, which climb only about 100 meters a day [50], and they probably had less dorsiflexed ankles when climbing [23]. The most likely interpretation of these data is that the Sterkfontein hominins loaded their distal tibiae using human-like ankle angles, hence a relatively extended lower limb posture. This interpretation is also supported by previous studies of the external morphology of StW 358, 389 and 567 [23], [24], which showed that in these individuals the loading of the ankle, the angle between the long axis of the tibia and the ankle joint surface, and the ankle range of motion were all humanlike, thus implying humanlike kinematics of the lower extremity during walking.

One limitation of our study was that we measured tarsal joint angles only while walking and not during running, which produces higher stresses and could contribute to the signal affecting the PTO. Chimpanzees, however, are almost solely knuckle walkers and rarely locomote bipedally, let alone run [46]. Similarly, there is no evidence or indication that australopiths ran habitually [51]. Therefore, out of the 3 species we studied, only humans sometimes run long distances. We had 6 human distal tibiae (Table S1). Three samples were Peruvian farmers (South America) and 3 samples are of unknown origin but have been in the Peabody Museum collection for many years, long before recreational running became common. The distal tibiae average PTO in all six human samples was 90.0°±2.3, indicating that loading patterns in all six samples were nearly identical. Given the likelihood that our comparative human samples did not come from individuals who frequently ran long distances it is reasonable to hypothesize that the day-to-day signals these bones were subjected to, and which they reflect, were primarily walking. Furthermore, peak ground reaction forces at the tarsal joint while running will be achieved in a more flexed (bent) joint angle (by about 15°) [52]. If these peak forces had a strong influence on the PTO, we should have seen a much lower difference in the distal tibiae PTO between humans and chimpanzees (i.e. the differences between humans and chimps in tarsal joint angle and in the distal tibiae PTO would not correspond to each other). Yet our results indicate a difference of 10.3° in tarsal joint angle and a 7.7° difference in PTO. This very close overlap between the two parameters indicates that walking is the main determinant of PTO in our samples. However, the small difference in PTO in comparison to joint angle (∼2.6°) may imply that running did contribute some signal, which affected PTO as well. Further research should study the relative contribution of running and walking to the adaptation of trabecular bone.

Humans differ from chimpanzees not only in 2D- and 3D-PTO but also by having significantly lower values of Tb.N and connectivity density and significantly higher values of Tb.Th, Tb.Sp and DA (i.e. in humans trabeculae are more oriented in one direction) (Table 1). More trabeculae that are more connected and anisotropic helps joints withstand high loads from multiple directions [53]. This finding accords with evidence that chimpanzees load their ankles during climbing and other activities in a much greater range of orientations than humans [23], [54]. Interestingly, the trabecular bone properties of the distal tibiae of Sterkfontein fossil hominins fall between human and chimpanzee values in terms of trabecular orientation (DA), which is strongly affected by loading, but also in terms of parameters that have both genetic and environmental influences such as Tb.N, Tb.Sp, and ConnD (Table 1). These differences tentatively suggest that, like chimpanzees, early hominins may have loaded their ankles in more diverse and intensive ways than modern humans.

It is worth comparing our results to those of a recent study that compared the trabecular architecture of the talus in humans, several non-human primates and australopiths [55]. Since the talus articulates with the distal tibia, one expects these two components of the ankle joint to be similar in their trabecular response to joint loading orientation. Although DeSilva and Devlin [55] also found that humans have much higher degree of anisotropy than chimpanzees and other non-human primates, they did not measure and compare PTO among species. Further, while DeSilva and Devlin found that chimps have significantly higher BV/TV and that australopiths are human-like in most respects, they did not find any other unique architectural differences such as ConnD, Tb.Th or Tb.N between humans, non-human primates and australopiths. Several differences between this study and DeSilva and Devlin's analysis likely account for the different findings. First, while DeSilva and Devlin looked at the entire trabecular volume of the talus, we analyzed VOIs just deep to the joint surface, where the signal of loading orientation is the strongest and clearest [44], [45]. In addition, rather than dividing the bone in to four quarters, our VOI's were specifically located deep to the cortical contact points with the talus, ensuring the measurement of directly loaded trabeculae, and avoiding the problem of averaging signals from other less relevant parts of the joint that may diminish or cancel any signal from variations in how the joint was loaded. Finally, DeSilva and Devlin used low resolution medical CT scans of 1 mm for the fossils, but we used high resolution microCT scans (32.8 μm), which is necessary to accurately measure the thickness and orientation of trabeculae, many of which are less than 0.2–0.25 mm thick. Future analyses of trabecular orientation in VOIs just under the joint surface of the talus using sufficiently high resolution are predicted to yield similar results to those reported here for the distal tibia.

A recent step in this direction is Su et al. 's (2013) [56] study of trabecular bone structure just deep to the talar trochlea of humans, non-human primates and a fossil sample of an extinct hominin dated around 1.6 million years ago. This fossil, KNM-ER 1464, although much younger than the two *Au. africanus* specimens presented here, is still a useful comparison because both studies try to correlate trabecular structure just deep to the joint surface to locomotion behavior of extant primates and by that to infer the locomotion behavior of extinct hominin taxa. Su et al. (2013) found that the PTO of talar trabecular structure just deep to the joint surface in the extinct hominin sample was similar to modern humans but strikingly different from African great apes (namely chimpanzee, gorilla and orangutan). Their results further support our findings that PTO is a potent and sensitive parameter to deduce locomotion behavior of extinct taxa.

There is no question that locomotor behavior must have varied among different species of *Australopithecus* given evidence for postcranial differences between the three best-known species, *Au. afarensis*, *Au. africanus*, and *Au sediba* as well as other Pliocene hominins such as the Burtele foot [57], [58]. MicroCT data from the distal tibia from these species as well as *Ardipithecus* and other early hominin taxa are needed to gain a better understanding of the range of variation in ankle angles during the evolution of hominin locomotion. Even so, evidence for habitually extended hindlimb postures in *Au. africanus* and whatever species is represented by StW 567 is significant because BHBK walking incurs a substantially higher cost of transport compared to the more extended posture used by humans due to higher moments around the knee and hip that must be countered by the large extensor muscles that cross these joints [19], [20], [59]. It is already well established that species of *Australopithecus* had some form of medial longitudinal arch capable of stiffening the foot for efficient toe-off (evident from the angle between the proximal and distal metatarsal ends to the diaphysis), hip abductors with a high mechanical advantage, and in some species, such as *Au. afarensis*, a calcaneus capable of resisting the impact forces caused by heel strike during walking [13], [60], [61]. In light of such adaptations, it is unsurprising that efficient, humanlike walking evolved in *Australopithecus* prior to the genus *Homo*.

## Materials and Methods

### Chimpanzee kinematics

Chimpanzee kinetics and kinematics were collected in 2005 and described previously [19], [20]. Three adult chimpanzees (two males and one females; mean age, 12 years; range, 6–18 years) walked quadrupedally at a Froude number of approximately 0.3 (1.2 m/s ±0.1) down a 10-m track equipped with an embedded force-plate (Kistler, Amherst, NY). We used data from chimpanzees during quadrupedal walking because this type of locomotion comprises more than 98% of their locomotion behavior [46] and hence will be the key determining factor for trabecular orientation in the distal tibiae. Vertical GRFs were measured using the force-plate at 1 kHz and normalized to body weight. Simultaneously, kinematic data were collected via high-speed video (125 frames/s; Redlake) with the hip, knee, ankle and foot marked on each subject using nontoxic water-based white paint. Trials were accepted only if the hindlimb contacted the force-plate cleanly and if fore-aft GRFv traces indicated constant forward speed (<10% difference between anterior and posterior impulse). Force-plate and kinematic data were smoothed using a zero-lag 4th order low pass Butterworth filter (cut-off frequencies were 12 Hz and 200 Hz for the kinematic and force-plate data, respectively). Ankle angle was measured in lateral view as the angle between two lines: from the lateral epicondyle of the femur to the lateral malleolus; from the posterior tuber calcaneus to the distal head of the 5th metatarsal (Fig. 1c). Because GRFv traces from some chimpanzee trials had a double force peak, tibia orientation relative to the long axis of the foot (ankle angle) was averaged over the period of stance when GRFv was greater than 75% of body weight (Fig. 1c). The chimpanzees were socially housed in large, outdoor enclosures at a United States Department of Agriculture registered and approved facility. Institutional Animal Care and Use Committee approval was obtained before the beginning of the study, and institutional animal care guidelines were followed throughout.

### Human kinematics

Six adult humans (3 males and 3 females; mean age, 33 years; range, 20–48 years) were measured while walking on custom-built, dual-belt, force instrumented treadmill (Bertec Corporation, Columbus OH, USA). Vertical GRFs were measured at 1000 Hz and normalized to body weight. Simultaneously, kinematic data were collected with an 8-camera Oqus kinematics system (Qualysis, Gothenburg, Sweden) at 500 Hz with markers on the lateral aspect of the hip, knee, ankle, and 5th metatarsal head. Subjects were recorded at a Froude number of 0.3 (1.3 m/s ±0.2) while walking with a normal gait and after walking for 2–4 minutes in a bent-hip bent-knee gait. We recorded humans BHBK walking to test whether ankle joint angle at midstance differs significantly from quadrupedal chimpanzees; these data would be important in the case that *Australopithecus africanus* distal tibia trabecular bone PTO differs significantly from humans but not chimpanzees. Ankle angle was measured in lateral view as the angle between two lines: from the lateral epicondyle of the femur to the lateral malleolus, and from the posterior tuber calcaneus to the distal head of the 5th metatarsal (Fig. 1a, b). Because GRFv traces from human trails had a double force peak, tibia orientation relative to the long axis of the foot (ankle angle) was averaged over the period of stance when GRFv was greater than 75% of body weight (Fig. 1). Experimental protocol was approved by Harvard University Committee on the Use of Human Subjects, and prior written informed consent was obtained from all subjects.

### Human and chimpanzee distal tibiae microCT scanning

Tibiae from adult *Homo sapiens* (n = 6) bones were obtained from the Peabody Museum of Archeology and Ethnology, Harvard University, Cambridge MA, USA. Adult *Pan troglodytes* (n = 6) tibiae were obtained from the Museum of Comparative Zoology, Harvard University, Cambridge MA, USA (Table S2). Chimpanzee tibiae are from wild-shot individuals from populations in West Africa. All bones had no traces of bone pathology. The distal part of all tibiae were microCT scanned at the Center for Nanoscale Systems, Harvard University using a Metris X-Tek HMX ST 225 scanner (Nikon Metrology Inc.) at 70 kV and 130 μA with no filter. Scan resolutions are summarized in Table S2. The output raw data (3142 projections, no frame averaging, and detector size 2000×2000 pixels) were imported into CT PRO software (Nikon Metrology Inc.) and reconstructed into 3D volumes.

### Fossil hominin distal tibiae microCT scanning

Three Sterkfontein tibiae (StW 358, 389, 567) were obtained from a collaborative project between the Department of Human Evolution, Max Planck Institute for Evolutionary Anthropology and the University of the Witwatersrand, South Africa, through its Institute for Human Evolution (Table S2), (we thank the Institute for Human Evolution at Witwatersrand University (Johannesburg) for allowing CT-scanning of the fossil material). These fossils are in an excellent state of preservation. Sample StW 389 has almost 4 cm of its diaphysis intact, StW 358 has around 1 cm of his diaphysis intact and StW 567 comprises only the most distal part of the tibia (Fig. 4). The entire 3D trabecular structure deep to the joint surface of all three tibiae is intact and detectable using an X-ray source (see Fig. 2d, 2e and 2f for sagittal views and Fig. 4). StW 358 has a crack running from the middle of the lateral edge to the middle of the anterior edge of the bone. Sample StW 389 is missing its medial condyle. Sample StW 567 is missing the postero-lateral corner of the distal tibia (Fig. 4). None of these missing or damaged areas were in the VOIs we analyzed. The fossils were microCT scanned in Johannesburg by the Department of Human Evolution, Max Planck Institute for Evolutionary Anthropology using a BIR ACTIS 225/300 high resolution scanner at 130 kV and 100 mA using a 0.5 brass filter. Scan resolutions are given in Table S2. The scans (2500 projections, three-frame averaging, and detector size 2048×2048 pixels) were reconstructed directly into 16-bit TIFF image stacks.

### Image processing

All reconstructed scans were imported into VGStudio Max 2.1 (Volume Graphics GmbH, Heidelberg Germany) and were reoriented along the long axis of the bone using the tibiae distal diaphysis and additional anatomical landmarks. All scans from the same species were superimposed to ensure identical orientation for all the bones (Fig. S1). The reconstructed scans were then cropped and saved as 16-bit TIFF image stacks. Each scan was saved as 16-bit TIFF image stacks twice, along two different axes: along the transverse plane (proximodistal) and along the sagittal plane (craniocaudally). The transverse image stacks were used to quantify principal orientations of trabeculae in 3D; the sagittal image stacks were used to quantify principal orientations of trabeculae in 2D in the sagittal plane (Figure 2). After cropping, image stacks were segmented (binarized) to differentiate bone from non-bone pixels using an edge-detection ray-casting algorithm (RCA) [62]. The RCA algorithm is advantageous over other conventional threshold detection techniques because it uses the gray level gradient of the image rather than the absolute gray-level values. Finally, images were converted into 8-bit TIFF image stacks (black pixels equal to “0” and white pixels equal to “255”).

### Trabecular bone properties and orientation calculation

Analyses of trabecular bone properties and PTO were performed using CTAn (CTAnalyzer; SkyScan, Belgium) [63]. Two spherical VOIs were selected within the trabecular bone of each tibia, one at the medial and one at the lateral distal articular surface of the tibia (tibial plafond), just deep to the cortex of the joint surface (i.e. proximal to the joint cortex and distal to the growth plate). The VOI's were positioned distally to the growth plate, in the epiphyses (secondary ossification center). Exact VOIs locations were chosen just deep to the contact points with the distal tibia (Fig. 3). VOI diameter was 200 pixels (for PTO calculation) and 400 pixels (for all other trabecular bone properties calculations) and varied in absolute size between species (i.e. VOI's absolute size was larger in humans; see Table S2 for scan resolutions). For orientation detection, VOI size was determined to be big enough to optimize the number of trabeculae that are near the joint surface following Harrigan [64], but not too big to avoid trabeculae more than 5–7 mm deep to the joint surface, which are less affected by the orientation of stresses in the joint. The following trabecular bone parameters were measured in 3D for the distal tibiae VOIs [63]: bone volume fraction (BV/TV), trabecular number (Tb.N), trabecular thickness (Tb.Th), trabecular separation (Tb.Sp), degree of anisotropy (DA) and connectivity density (ConnD). DA measures trabecular alignment along a preferred axis; a larger value indicates a stronger tendency of the trabecular structure to align itself along a preferred orientation. ConnD defines how many connections per mm^3^ between different trabeculae can be severed before the trabecular tissue will be divided into two separate parts. PTO was determined by CTAn software using the mean intercept length (MIL) technique. The MIL technique superimposes a linear grid over a selected area (in 2D) or volume (in 3D) and counts the number of intersections between the grid and the bone/non-bone interface. The “mean intercept length” is defined as total line length divided by the number of intersections [65]. By rotating the grid's orientation by a constant angle (ω) and measuring the MIL at each angle, it is possible to determine the orientation at which the MIL is the largest (i.e., has the fewest intersections between bone and non-bone pixels). The output eigenvector values (x,y and z coordinates of the principle orientation vector situated on the surface of the spherical VOI) were imported into stereographic projection software (StereoNett, Institute of Geology, Ruhr University, Bochum, Germany) and were visualized using an equal-angle stereoplot (A stereoplot is a 2D map which is created by projecting points from a surface of a sphere to a tangential plane, Fig. 3a). 2D-PTO in the sagittal plane was also determined for each bone using the MIL technique from 10 sagittal slices in the middle of each VOI (Fig. 2). Note that 2D-PTO and ankle angle are shifted consistently by approximately 8° because 2D-PTO was measured relative to a plane perpendicular to the long axis of the tibia, not the surface of the joint; for similar shifts, see also Pontzer 2006 [44], Barak 2011 [45].

### Statistical Analyses

Statistical analyses were performed using R, version 2.15.0 (R Foundation for Statistical Computing, Vienna, Austria; www. r-project.org). Values given are mean and standard deviations (S.D) unless indicated differently. Statistical significance was determined using 95% confidence intervals. Statistically significant differences between species for trabecular bone parameters other than orientation were determined using Wilcoxon rank-sum test (Table 1). In order to test if PTO differed significantly between groups we ran a permutation test. This method allocates the data points into two new groups, and then uses a non-parametric t-test to test if the original groups differ significantly or not. This cascade is repeated until the entire possible population of groups were created and tested (we thank the Institute for Quantitative Social Science at Harvard University (and especially Steven Worthington) for help in performing the permutation tests). For the sagittal plane measurements of 2D-PTO, we determined the medians of the groups and tested them against the median of all other possible allocated groups using the same original datapoints. For the 3D-PTO, we calculated each group's centroid on the surface of the sphere (VOI) using the haversine formula, which calculates the shortest distance between two points on a surface of a sphere. We then measured the distance between the centroids of the two original groups. Finally, we executed a permutation test, checking the measured distance against the distance between centroids of all other possible allocated groups (using the same datapoints). A P-value ≤0.05 indicates that the distance between the two group centroids is significant. In one permutation test (comparing the chimpanzees to humans and early hominins in the medial 3D VOI) we removed one chimpanzee outlier (see Fig. 3c; point MCZ 10736: longitude 91.5 and latitude 69.3), including this outlier yields a P-value of 0.07.

## Supporting Information

Figure S1 **The tibiae distal surfaces of Chimpanzees, humans and early hominin fossils visualized using VGStudio Max 2.1.** Using VGStudio Max 2.1 bones were reoriented along their long axis. Next, all bone scan reconstructions from the same species were overlapped in 3D to ensure identical orientation. Each illustration shows a combination of all bones from the same group superimposed one on top of the other: chimpanzees (a), humans (b) and early hominin fossils (c). In view is the tibiae distal surfaces (tibial plafond), the medial malleolus is at the upper right side of each illustration.
(TIF)

Table S1 **Longitude and latitude coordinates for the 3D-PTO presented in** **Fig. 3b and 3c** **.**
(DOCX)

Table S2 **A list of all samples used.**
(DOCX)
