# Supplementary figures and images for: Trabecular Evidence for a Human-Like Gait in Australopithecus africanus
Source: PLoS One. 2013 Nov 5;8(11):e77687. doi: 10.1371/journal.pone.0077687 (PMC3818375; doi:10.1371/journal.pone.0077687)

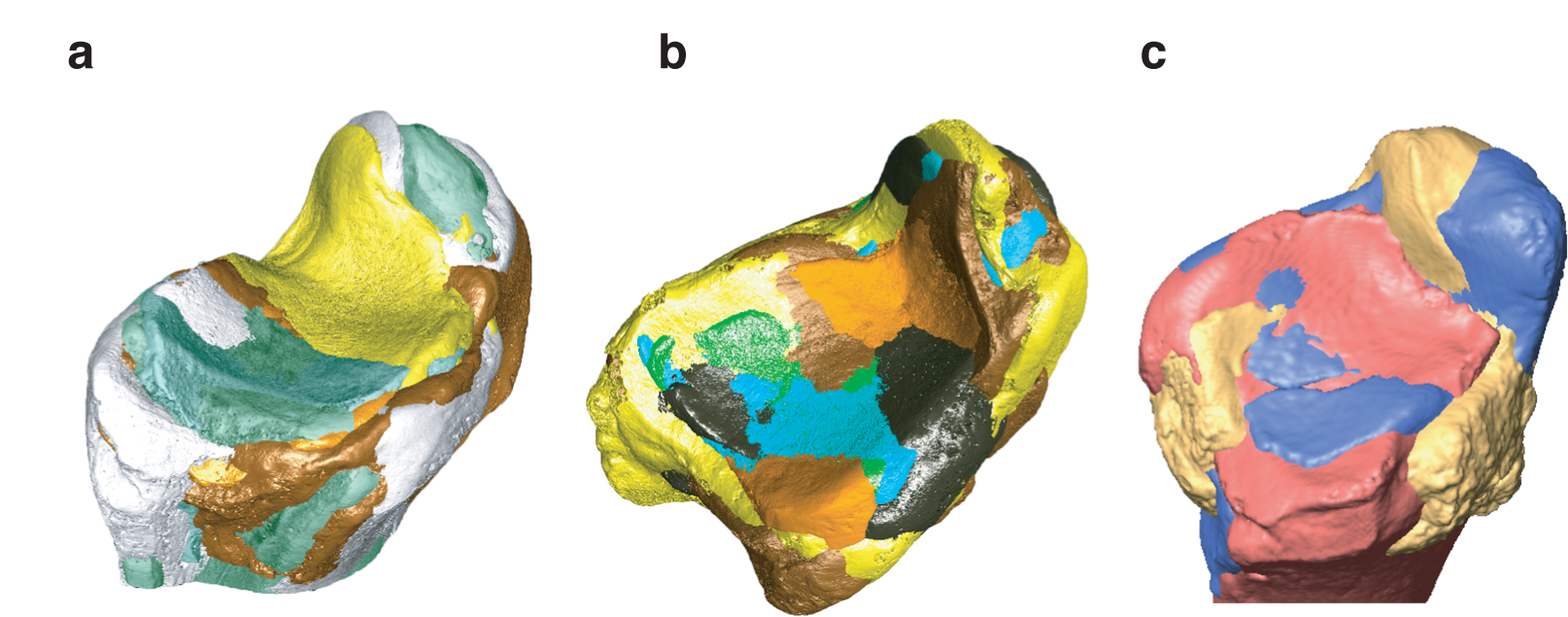

Supplement: Figure S1 — The tibiae distal surfaces of Chimpanzees, humans and early hominin fossils visualized using VGStudio Max 2.1. Using VGStudio Max 2.1 bones were reoriented along their long axis. Next, all bone scan reconstructions from the same species were overlapped in 3D to ensure identical orientation. Each illustration shows a combination of all bones from the same group superimposed one on top of the other: chimpanzees (a), humans (b) and early hominin fossils (c). In view is the tibiae distal surfaces (tibial plafond), the medial malleolus is at the upper right side of each illustration. (TIF) [file pone.0077687.s001.tif]
