# Supplementary material for: Trabecular Evidence for a Human-Like Gait in Australopithecus africanus
Source: PLoS One. 2013 Nov 5;8(11):e77687. doi: 10.1371/journal.pone.0077687 (PMC3818375; doi:10.1371/journal.pone.0077687)
Supplement: Table S1 — Longitude and latitude coordinates for the 3D-PTO presented in Fig. 3b and 3c . (DOCX) [file pone.0077687.s002.docx]

**Table S1**
Longitude and latitude coordinates for the 3D-PTO presented in Fig. 3b and 3c.

|  | | Medial | | Lateral | |
| --- | --- | --- | --- | --- | --- |
|  |  | longitude λ | latitude φ | longitude λ | latitude φ |
| *Pan troglodytes* | MCZ 6244 | 61.5 | 80.2 | 148.7 | 84.5 |
| (Chimpanzee) | MCZ 10736 | 91.5 | 69.3 | 79.8 | 72.6 |
|  | MCZ 23164 | 48.4 | 80.4 | 103.7 | 72.3 |
|  | MCZ 19187 | 63.3 | 77.7 | 102.0 | 80.8 |
|  | MCZ 15312 | 44.9 | 76.0 | 287.7 | 87.3 |
|  | MCZ 20041 | 49.1 | 79.1 | 85.0 | 76.9 |
| H. sapiens | PM 33-81-00 /N868.0.1 | 107.5 | 84.9 | 354.1 | 87.1 |
| (Human) | PM 39-2-00/N3457.0 | 84.4 | 79.9 | 66.2 | 83.8 |
|  | PM 33-81-00 /N1067.0 | 92.9 | 82.3 | 94.1 | 89.1 |
|  | PM 80-61-30/58801.0.1 | 87.1 | 77.8 | 295.9 | 88.5 |
|  | PM 16-5-30/59382.0.1 | 42.5 | 85.6 | 25.1 | 82.3 |
|  | PM 16-5-30/59379.0.1 | 21.0 | 82.9 | 29.7 | 85.2 |
| Au. africanus | StW 358 | 93.6 | 81.1 | 47.3 | 87.6 |
|  | StW 389 | 74.7 | 73.2 | 345.3 | 81.7 |
| Homo sp. indet. | StW 567 | 86.4 | 81.2 | 112.9 | 85.6 |

The longitude and latitude were derived from the eigenvector x, y and z coordinates which are the intersection point between the eigenvector and the surface of the spherical VOI.

MCZ, Museum of Comparative Zoology, Harvard University

PM, Peabody Museum, Harvard University
StW, Sterkfontein, South Africa
