# Supplementary material for: Trabecular Evidence for a Human-Like Gait in Australopithecus africanus
Source: PLoS One. 2013 Nov 5;8(11):e77687. doi: 10.1371/journal.pone.0077687 (PMC3818375; doi:10.1371/journal.pone.0077687)
Supplement: Table S2 — A list of all samples used. (DOCX) [file pone.0077687.s003.docx]

**Table S2**

Samples Used

| **species** | **sample #** | **sample origin** | **collection** | **scanned @** | **microCT scanner** | **resolution (mm)** |
| --- | --- | --- | --- | --- | --- | --- |
| *Homo sapiens* | 33-81-00 /N868.0.1 | forensic, transfer from Warren Anatomical museum | PM | Harvard U. | Metris X-Tek HMX ST 225 | 0.036 |
| *Homo sapiens* | 39-2-00/N3457.0 | No info | PM | Harvard U. | Metris X-Tek HMX ST 225 | 0.036 |
| *Homo sapiens* | 33-81-00 /N1067.0 | forensic, transfer from Warren Anatomical museum | PM | Harvard U. | Metris X-Tek HMX ST 225 | 0.036 |
| *Homo sapiens* | 16-5-30/59379.0.1 | South America, Peru, La Libertad, Incasic cave | PM | Harvard U. | Metris X-Tek HMX ST 225 | 0.036 |
| *Homo sapiens* | 16-5-30/59382.0.1 | South America, Peru, La Libertad, Incasic cave | PM | Harvard U. | Metris X-Tek HMX ST 225 | 0.036 |
| *Homo sapiens* | 80-61-30/58801.0.1 | South America, Peru, ancient grave | PM | Harvard U. | Metris X-Tek HMX ST 225 | 0.036 |
| *Pan troglodytes* | 15312 | Africa, Cameroon, Lolodorf | MCZ | Harvard U. | Metris X-Tek HMX ST 225 | 0.0259 |
| *Pan troglodytes* | 19187 | Africa, Cameroon, Sanaga Maritime, Sakbayeme | MCZ | Harvard U. | Metris X-Tek HMX ST 225 | 0.025 |
| *Pan troglodytes* | 20041 | Africa, Cameroon, Sanaga Maritime, Sakbayeme | MCZ | Harvard U. | Metris X-Tek HMX ST 225 | 0.025 |
| *Pan troglodytes* | 23164 | Africa, Cameroon, Sanaga Maritime, Sakbayeme | MCZ | Harvard U. | Metris X-Tek HMX ST 225 | 0.025 |
| *Pan troglodytes* | 10736 | Africa, West Africa | MCZ | Harvard U. | Metris X-Tek HMX ST 225 | 0.0267 |
| *Pan troglodytes* | 6244 | Africa, West Africa | MCZ | Harvard U. | Metris X-Tek HMX ST 225 | 0.025 |
| Homo sp. indet | StW 567 | South Africa, Sterkfontein; Member 5 East | WITS | MPI-EVA | BIR ACTIS 225/300 | 0.0328 |
| *Australopithecus africanus* | StW 358 | South Africa, Sterkfontein; Member 4 | WITS | MPI-EVA | BIR ACTIS 225/300 | 0.0328 |
| *Australopithecus africanus* | StW 389 | South Africa, Sterkfontein; Member 4 | WITS | MPI-EVA | BIR ACTIS 225/300 | 0.0328 |

PM, Peabody Museum, Harvard University

MCZ - Museum of Comparative Zoology

WITS - University of Witwatersrand, Johannesburg, South Africa

MPI-EVA - Max Planck Institute for Evolutionary Anthropology

Note: Chimpanzees samples 10736 and 6244 were collected during 1917 and 1879 respectively and have no additional and more specific location data beside “West Africa”.
